# Nutrient Intake and Its Association with Appendicular Total Lean Mass and Muscle Function and Strength in Older Adults: A Population-Based Study

**DOI:** 10.3390/nu16040568

**Published:** 2024-02-19

**Authors:** Miguel Germán Borda, Jessica Samuelsson, Tommy Cederholm, Jonathan Patricio Baldera, Mario Ulises Pérez-Zepeda, George E. Barreto, Anna Zettergren, Silke Kern, Lina Rydén, Mariana Gonzalez-Lara, Salomón Salazar-Londoño, Gustavo Duque, Ingmar Skoog, Dag Aarsland

**Affiliations:** 1 Centre for Age-Related Medicine (SESAM), Stavanger University Hospital, 4011 Stavanger, Norway; mmborda@gmail.com (M.G.B.); jonathan_patricio@javeriana.edu.co (J.P.B.); daarsland@gmail.com (D.A.); 2 Semillero de Neurociencias y Envejecimiento, Ageing Institute, Medical School, Pontificia Universidad Javeriana, Bogotá 110231, Colombia; salazarl.salomon@javeriana.edu.co; 3 Division of Clinical Geriatrics, Center for Alzheimer Research, Department of Neurobiology, Care Sciences and Society, Karolinska Institutet, 14186 Stockholm, Sweden; 4 Institute of Neuroscience and Physiology, Sahlgrenska Academy, University of Gothenburg, 41345 Gothenburg, Sweden; jessica.samuelsson@neuro.gu.se (J.S.); anna.zettergren@neuro.gu.se (A.Z.); silke.kern@neuro.gu.se (S.K.); lina.ryden@gu.se (L.R.); ingmar.skoog@neuro.gu.se (I.S.); 5 Department of Public Health and Caring Sciences, Clinical Nutrition and Metabolism, Uppsala University, 62167 Uppsala, Sweden; tommy.cederholm@pubcare.uu.se; 6 Theme Inflammation & Aging, Karolinska University Hospital, 14186 Stockholm, Sweden; 7 Escuela de Estadística, Universidad Autónoma de Santo Domingo, Santo Domingo 10103, Dominican Republic; 8 Instituto Nacional de Geriatría, Dirección de Investigación, Mexico City 10200, Mexico; mperez@inger.gob.mx; 9 Centro de Investigación en Ciencias de la Salud (CICSA), Facultad de Ciencias de la Salud, Universidad Anáhuac México Campus Norte, Huixquilucan 52786, Mexico; 10 Department of Biological Sciences, University of Limerick, V94 PH61 Limerick, Ireland; george.barreto@ul.ie; 11 Department of Psychiatry, Cognition and Old Age Psychiatry, Sahlgrenska University Hospital, SE-413 45 Mölndal, Sweden; 12 Faculty of Health, Dalhousie University, Halifax, NS B3H 4R2, Canada; mariana.glara@gmail.com; 13 Dr. Joseph Kaufmann Chair in Geriatric Medicine, Department of Medicine, McGill University, Montreal, QC H3A 2B4, Canada; 14 Bone, Muscle & Geroscience Group, Research Institute of the McGill University Health Centre, Montreal, QC H4A 3J1, Canada; 15 Department of Old Age Psychiatry, Institute of Psychiatry, Psychology, and Neuroscience, King’s College London, London SE1 9RT, UK

**Keywords:** sarcopenia, body composition, aging, diet, walking speed, muscle strength

## Abstract

Treatment options for sarcopenia are currently limited, and primarily rely on two main therapeutic approaches: resistance-based physical activity and dietary interventions. However, details about specific nutrients in the diet or supplementation are unclear. We aim to investigate the relationship between nutrient intake and lean mass, function, and strength. Data were derived from the Gothenburg H70 birth cohort study in Sweden, including 719,70-year-olds born in 1944 (54.1% females). For independent variables, the diet history method (face-to-face interviews) was used to estimate habitual food intake during the preceding three months. Dependent variables were gait speed (muscle performance), hand grip strength (muscle strength), and the appendicular lean soft tissue index (ALSTI). Linear regression analyses were performed to analyze the relationship between the dependent variables and each of the covariates. Several nutrients were positively associated with ALSTI, such as polyunsaturated fatty acids (DHA, EPA), selenium, zinc, riboflavin, niacin equivalent, vitamin B12, vitamin D, iron, and protein. After correction for multiple comparisons, there were no remaining correlations with handgrip and gait speed. Findings of positive correlations for some nutrients with lean mass suggest a role for these nutrients in maintaining muscle volume. These results can be used to inform clinical trials to expand the preventive strategies and treatment options for individuals at risk of muscle loss and sarcopenia.

## 1. Introduction

Aging is intricately associated with the progressive decline in muscle mass and strength along with physical function; a phenomenon commonly referred to as sarcopenia [1]. This deterioration tends to intensify with age and is exacerbated by factors such as a sedentary lifestyle, weight loss, and physical illnesses. Importantly, the repercussions of muscle mass loss and sarcopenia extend beyond mere physiological changes and are linked to a spectrum of adverse health outcomes among older adults, including an elevated risk of falls, increased mortality, cognitive decline, and heightened vulnerability to disability [2,3]. Sarcopenia is one of several conditions that accompany the aging process, some of which are not fully described or comprehended, given that population aging is a relatively recent phenomenon in clinical medicine. These conditions typically interact with each other, elevating the individual risk of each and, more significantly, amplifying the likelihood of adverse outcomes [4]. Among these conditions are cognitive decline, frailty, late-life depressive symptoms, and malnutrition, to name a few.

Notably, sarcopenia has emerged as a precise prognostic biomarker for various chronic conditions, spanning from liver cirrhosis, diabetes, osteoporosis, chronic obstructive pulmonary disease, and kidney failure to cancer, as well as acute events such as stroke and trauma [5]. The prevalence of sarcopenia has been observed to rise with age, with recent reports indicating an impact on 10% to 16% of the global older adult population. Interestingly, its prevalence is notably higher among older adults with chronic diseases compared to the general population, ranging from 18% in individuals with diabetes to a staggering 66% in those diagnosed with cancer [6].

Risk factors for sarcopenia include aging, a sedentary lifestyle, chronic diseases, hormonal changes, and inadequate nutrition. The etiopathology involves a complex interplay of factors such as inflammation, oxidative stress, and hormonal imbalances, culminating in the dysregulation of muscle protein synthesis and breakdown [6]. Diagnosing sarcopenia often relies on criteria combining measurements of muscle mass, strength, and physical performance. Various assessment tools, including dual-energy X-ray absorptiometry (DXA) and bioelectrical impedance analysis (BIA), aid in determining muscle mass. Grip strength and gait speed are commonly used indicators of muscle strength and physical performance [1], respectively.

Treatment strategies encompass exercise, particularly resistance training, and adequate nutritional support, with an emphasis on protein intake [7]. Rehabilitation programs aim to improve mobility and functionality. Despite the evident health benefits, sarcopenia poses substantial economic challenges to countries, with increased healthcare costs for diagnosis, treatment, and rehabilitation. Moreover, the societal burden is exacerbated by indirect costs associated with disability, loss of productivity, and the need for long-term care, underlining the importance of preventive measures and early intervention to mitigate the financial impact of sarcopenia [8].

While physical exercise and elevated protein intake have demonstrated effectiveness in reducing sarcopenia, there is insufficient evidence to support the effectiveness of alternative treatment options, such as medical foods or nutritional interventions. Therefore, specific dietary recommendations cannot be made [9]. Certain nutrients, including vitamin D, calcium, magnesium, selenium, iron, zinc, and omega-3 fatty acids (or their combination), may have a positive impact on muscle maintenance and strength [10,11], with larger increases being reported in muscle quality and function for women compared to men for nutrients like long-chain n-3 polyunsaturated fatty acids (PUFAs) [12]. However, the study of dietary patterns based on real-world data needs to be augmented to better understand their association with sarcopenia at a structural and functional level.

Therefore, it is crucial to develop a comprehensive understanding of the interplay between dietary components and their correlation with appendicular and total lean mass, gait speed (a marker of muscle performance), and hand grip strength (indicative of muscle strength). The present study aims to delve into the relationship between dietary components and various aspects of muscle health to provide valuable insights that could guide future interventions. The overarching hypothesis propelling this investigation is that a higher consumption of specific dietary elements may be linked to increased appendicular and total lean mass, faster gait speed, and stronger hand grip among older adults residing in the community.

## 2. Materials and Methods

### 2.1. Design, Settings, and Participants

Cross-sectional data were derived from the Swedish population-based Gothenburg H70 Birth Cohort Study (H70 study) on 70-year-olds born in 1944 and examined between 2014 and 2016 (n = 1203). The response rate was 72% [13,14]. See a detailed sample description in Supplementary Materials Figure S1.

### 2.2. Anthropometry

We assessed body weight and height using a calibrated electronic scale and a stadiometer, respectively. Body composition analysis was conducted through DXA scanning using the Lunar Prodigy scanner. The Appendicular Lean Soft Tissue Index (ALSTI) was employed to estimate lean mass, calculated by dividing the sum of lean soft tissue in the arms and legs by the square of body height.

### 2.3. Muscle Strength and Performance

The physical performance tests encompassed self-selected and maximum gait speed assessments, quantified in meters per second across a 30-m indoor distance, and initiated from a standing position [15,16].

Additionally, the distance covered in a six-minute indoor walking test was recorded in meters. Grip strength was assessed using both a Martin Vigorimeter and, for a subset of participants, a JAMAR dynamometer, with careful attention to maintaining the shoulder joint in a neutral position. This evaluation was performed three times for each hand, and the highest recorded value from the dominant hand was taken as the conclusive result [14]. These grip strength tools have been extensively validated and demonstrated reliability in the geriatric population [17]. Results are reported in kPa as provided by the study and as previously reported by Wallengren et al. [18].

Patients were divided into probable, confirmed, and severe sarcopenia based on the 2019 European consensus [1]. Low muscle strength defines sarcopenia as probable, and this with low muscle quantity or quality defines sarcopenia as confirmed. A diagnosis of severe sarcopenia is based on a confirmed disease with a low physical performance.

### 2.4. Dietary Examination

The previously validated diet history method (DH) was used to estimate the dietary intake [19]. The dietary history DH employed in this study involved a semi-structured face-to-face interview conducted by a registered dietitian, aiming to estimate the habitual dietary intake over the preceding three months. The interview protocol included a meal-pattern interview, supplemented by a food list with inquiries about the typical frequencies and portion sizes of consumed foods. The diet history interview comprises 34 questions. Notably, each inquiry related to food groups (e.g., fish/meat/vegetables) encompasses multiple options, allowing participants to specify the exact type of milk product, bread, fish, etc., consumed, along with detailed information on portion sizes and cooking methods. The database from The Swedish Food Agency, with access to over 2400 distinct foods, was provided, including specific varieties of bread, meat, vegetables, and more. This extensive database enables a meticulous and comprehensive analysis of the detailed dietary information provided by participants. The recorded dietary intake was expressed as grams of food items typically consumed per day, week, or month using the nutritional calculation computer program Dietist Net Pro, which incorporates the SFA’s nutrient database from 2015. The estimated mean daily energy and nutrient intake were derived from the results obtained through the DH interview. It is important to note that dietary supplements were not considered in this study [19].

### 2.5. Dietary Components Included

Mean daily energy, alcohol, and nutrient intakes were estimated based on results from the DH interview. In this study, we included data on energy (mean kcal intake/day), protein, carbohydrates, dietary fiber, total fat, saturated fatty acids, monounsaturated fatty acids, PUFAs, eicosapentaenoic acid (EPA), docosahexaenoic acid (DHA), and alcohol intake, all measured in mean gram intakes/day. Nutrients included were vitamins C (mean mg intake/day), D (mean µg intake/day), E (mean mg intake/day), thiamine (B1) (mean mg intake/day), riboflavin (B2) (mean mg intake/day), niacin equivalents (mean mg intake/day), B6 (mean mg intake/day), B12 (mean µg intake/day), folate (B9) (mean µg intake/day), and retinol equivalents (mean µg intake/day), as well as iron (mean mg intake/day), calcium (mean mg intake/day), phosphorus (mean mg intake/day), magnesium (mean mg intake/day), potassium (mean mg intake/day), zinc (mean mg intake/day), and selenium (mean µg intake/day) [19].

### 2.6. Statistical Analysis

First, a description of the included variables was provided, estimating percentages for categorical variables and averages, as well as standard deviations for numerical variables. Subsequently, differences between men and women were assessed by applying a Pearson’s chi-square test for categorical variables and Student’s t-test for numerical variables. Finally, linear regression models were used to analyze the relationship between the dependent variables (i.e., ALSTI, handgrip strength, and gait speed) and each of the covariates while controlling for sex, mean energy intake (kcal/day), and the interaction between the exposure variable and sex. The estimated coefficients (Est), standard errors (SE), and *p*-values for each exposure variable were reported.

To measure the size of the effect of the studied associations, we utilized the Spearman’s Rho coefficient, assuming the following cutoff points: at least 0.8 (very strong), 0.6 up to 0.8 (moderately strong), 0.3–0.5 (fair), and <0.3 (poor) [20,21].

In addition, correlation graphs were constructed to visualize the association between the ALSTI and the statistically significant covariates. The statistical significance level was set at 0.05, and the Benjamini and Hochberg method was used to adjust for multiple comparisons [22]. The analyses were performed using R version 4.2.1 and STATA 17.0 software.

### 2.7. Ethical Considerations

Participants provided informed consent, and the study was approved by the Regional Ethical Review Board in Gothenburg with the number 869-13.

## 3. Results

### 3.1. Descriptive Results

A total of 719 individuals were included (56.7% females). Muscle measurements were significantly higher in men: ALSTI (kg/m^2^) mean for men and women were 7.83 ± 0.77 and 6.21 ± 0.64 (*p* < 0.001), respectively. Sarcopenia, confirmed, and Sarcopenia, severe, were more prevalent in men (Table 1).

Men had a predominantly higher nutrient intake, except for vitamin C (mg) and E (mg). No participants had an intake below the average requirement level (Table 2).

### 3.2. Association between Nutrients and ALSTI

**Figure 1:**
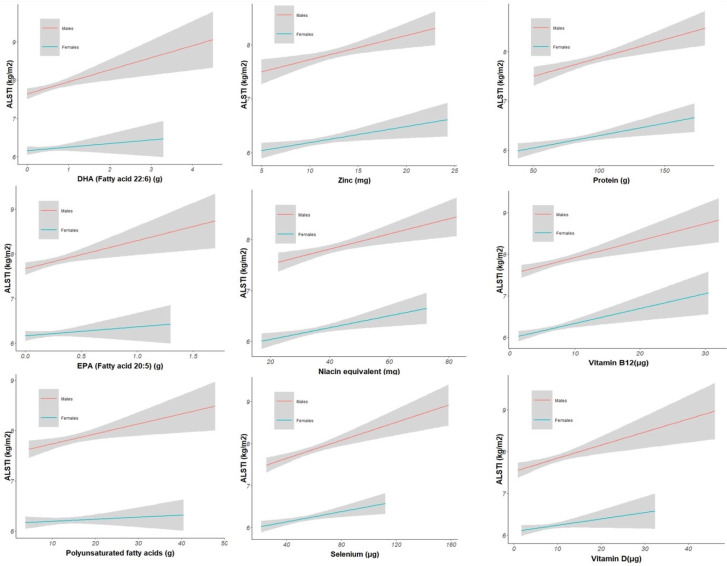
Correlation graphs between nutrients and ALSTI by sex. Fitted values with confidence intervals show the association. The deviation of the trend lines shows differences between men and women. Appendicular lean soft tissue index = ALSTI, Eicosapentaenoic acid = EPA, and Docosahexaenoic acid = DHA.

Several nutrients were positively associated with ALSTI, maintaining statistical significance after multiple comparison adjustments. The strongest associations were found for the PUFAs (Est 0.0171 SE 0.0072, *p* adjusted = 0.034, Spearman’s Rho = 0.190), EPA (Est 0.5892 SE 0.1968, *p* adjusted = 0.008, Spearman’s Rho = 0.117), DHA (Est 0.2978 SE 0.0875, *p* adjusted = 0.003, Spearman’s Rho = 0.125), selenium (Est 0.0109 SE 0.0024, *p* adjusted < 0.001, Spearman’s Rho =0.227), zinc (Est 0.0604 SE 0.0172, *p* adjusted = 0.003, Spearman’s Rho = 0.302), riboflavin (Est 0.2068 SE 0.0838, *p* adjusted = 0.029, Spearman’s Rho = 0.229), niacin equivalent (Est 0.0176 SE 0.0049, *p* adjusted = 0.003, Spearman’s Rho = 0.367), vitamin B12 (Est 0.0397 SE 0.0104, *p* adjusted = 0.001, Spearman’s Rho = 0.231), vitamin D (Est 0.0292 SE 0.0089, *p* adjusted = 0.004, Spearman’s Rho = 0.219), iron (Est 0.0439 SE 0.0137, *p* adjusted = 0.005, Spearman’s Rho = 0.240), and protein (Est 0.017 SE 0.0023, *p* adjusted < 0.001, Spearman’s Rho =0.330) (Supplementary Materials Table S1). Correlation graphs are displayed in Figure 1.

### 3.3. Association between Nutrients and Handgrip Strength (kPa)

Fibers, iron, vitamin E, and B6 were significantly related to handgrip strength. However, after multiple comparisons, none of them remained significant (Supplementary Materials Table S1).

### 3.4. Association between Nutrients and Gait Speed 30 m (m/s)

Gait speed correlated with the consumption of calcium and riboflavin; however, after multiple comparison adjustments, no variables remained significantly correlated (Supplementary Materials Table S1).

## 4. Discussion

The results of this study indicate that there are several nutrients associated with ALSTI; specifically, positive associations were identified for the intake of protein, PUFAs such as DHA and EPA, iron, vitamin D, riboflavin, niacin equivalents, vitamin B12, zinc and selenium. These nutrients are essential in supporting correct body functioning and contributing significantly to overall health and well-being [10,23]. Enhancing muscle health by increasing the consumption of these nutrients can potentially lead to benefits such as muscle mass growth, improved strength and function and enhanced overall health. Nutrient intake in relation to sarcopenia has been previously studied. The most widely studied association has been that inadequate protein intake is associated with a higher prevalence of sarcopenia, low muscle mass, and low muscle strength. Such findings have led to strong recommendations for adequate protein intake in older adults to prevent and treat sarcopenia [24,25].

Our findings highlight that some nutrients, e.g., vitamin D, correlate with a higher lean mass. Vitamin D can suppress the activity of atrophy-related transcription factors and may also stimulate protein synthesis [26]. A recent report by Yang et al. demonstrated that a combination of physical inactivity and low serum vitamin D levels can exacerbate muscle atrophy in older individuals [27]. However, the consensus does not recommend vitamin D supplementation in older adults with sarcopenia, as there is insufficient evidence to support its effectiveness [28,29,30].

There is growing interest in exploring the effects of dietary antioxidants such as vitamins E, C, CoQ10, and anthocyanins on age-related muscle loss and dysfunction. Antioxidants may positively affect muscle growth in the younger population [31]. For older adults, antioxidants have mainly been studied for cognitive effects, with conflicting results [32]. Several other micronutrients have been linked to sarcopenia. For example, there is evidence that low vitamin B12 (necessary to produce red blood cells and maintain nerve sheaths) is associated with muscle weakness and decreased physical function [33]. In our study, the intake of vitamin B12 was significantly associated with ALSTI. Of note, no participants in this cohort had an intake below the average requirement levels. Among older adults, vitamin B12 deficiency is usually not related to insufficient intake but rather to decreased uptake, due to atrophic gastritis or metformin intake, which are conditions that require vitamin B12 supplementation [34]. In addition, other minerals were significantly related to muscle mass in our sample. For example, selenium may be important for sarcopenia prevention [10]. It has been reported that selenium may play a role in preventing sarcopenia by reducing oxidative stress and inflammation, factors that likely contribute to the age-related loss of muscle mass [35].

Omega-3 fatty acids have anti-inflammatory properties and may help prevent muscle loss and maintain muscle function in older adults. Omega-3 PUFAs directly reduce the synthesis of cytokines that have pro-inflammatory functions [36]. In addition, omega-3 PUFAs, particularly DHA, are key components of the phospholipids that form cell membranes and preserve the integrity and function of the cells [24,36]. Smith et al. found that supplementation with DHA and EPA increased the muscle strength in older women [37]. It was also observed that supplementation with DHA and EPA in older adults increased muscle protein synthesis [38]. However, despite the growing evidence on this topic, additional research is needed to determine the precise dosage, frequency, and application of sarcopenia treatment and prevention [39].

Nutrients like EPA, DHA, Vitamin B12, and selenium have also been assessed to prevent cognitive decline in older adults [40]. It is proposed that a combination of nutrients might be required to synergistically increase brain levels of phosphatide molecules that comprise the bulk of brain synaptic membranes [41]. This might also have a role in the neuromuscular junction. In a pilot trial, at 12 weeks, significant improvement was noted in the delayed verbal recall task in the treatment group compared with the control [40].

Although our study provides valuable insights into the relationship between the muscle volume and dietary intake of several nutrients, it is important to acknowledge the limitations of this study. The study sample consisted of older individuals from a specific geographic area who also fulfilled average nutrient requirement levels, limiting the generalizability of the findings to other populations. Moreover, the study design was cross-sectional, making it challenging to establish the causality or determine the temporal sequence of events. Additionally, relying on self-reported data may introduce social desirability bias, memory recall bias, and other cognitive biases. However, this sample was relatively young and cognitively healthy, and the objective of this paper mainly focused on physical measures. Finally, the results of this paper were through diet history registry rather than blood nutrient levels.

Our analysis was conducted by separately examining two key aspects: muscle mass and muscle function. Muscle mass serves as a direct indicator linked to nutritional status, while the amalgamation of muscle mass and muscle function defines the condition known as sarcopenia. Analyzing these variables individually, owing to their linear nature, facilitates a more robust and dependable statistical approach. Notwithstanding, the intricate challenge of extrapolating the individual contribution of each nutrient to the muscle mass/strength is noteworthy. Many of these nutrients serve as cofactors/activators in various metabolic processes, with zinc being a prominent example. Caution is essential when drawing conclusions based on findings, given the multifaceted roles these nutrients play.

In the context of omega-3, which involves a more intricate metabolic process, these nutrients could enhance the metabolism of PUFAs to benefit muscle, particularly in males. Significant differences between males and females raise some questions [12]: could the estrogen stimulation of enzymes in this pathway explain the observed lower omega-3 levels in women over 70? Additionally, the decrease in circulating estrogen in postmenopausal women may impact the processing/synthesis of these nutrients, leading to suboptimal outcomes. These aspects warrant further investigation to enhance the depth of understanding regarding the observed patterns [42].

This study has several strengths. The substantial sample size enhances the statistical power for detecting associations. Additionally, the population-based cohort ensures a representation of the region’s population, supported by comprehensive clinical assessments. The utilization of DXA to measure the lean mass is a notable strength, considering it is widely recognized as a reference method.

Moreover, in contrast to previous studies that examined a broader age range [24], this study exclusively focuses on 70-year-old adults leading relatively healthy lives within the community. The age of 70 represents a critical stage where individuals may either embark on a trajectory of healthy aging by taking proactive measures to prevent diseases or, conversely, experience the onset, acquisition, or exacerbation of chronic conditions leading to disability. In addition, nutrient intake was not assessed through a questionnaire but via a more detailed, in-person interview conducted by a certified dietitian. Consequently, our methods enhance the reliability and homogenization of the results, contributing to a more nuanced understanding of the specific demographic under investigation [43].

It is important to note that our study not only considers muscle mass but also incorporates an assessment of muscle function. Additionally, our research presents a broader examination of nutrients compared to most available studies, which tend to focus on a single nutrient, e.g., protein, vitamin D, or fatty acids [44].

The results may be important with the potential to improve the management alternatives for sarcopenia. However, due to controversies and insufficient evidence in certain areas, such as vitamin D or omega-3 supplementation, further research is needed to determine the optimal dosage, frequency, and application of nutritional interventions for sarcopenia treatment and prevention.

## 5. Conclusions

Our analysis found that various nutrients, including fatty acids, zinc, and iron, were associated with higher muscle mass in cross-sectional examinations. These findings contribute significantly to the expanding body of knowledge concerning the role of specific nutrients in geriatric conditions. They underscore the potential for targeted interventions and strategies to promote healthy aging and preserve muscle health. Consequently, incorporating these nutrients into one’s diet or considering an appropriate supplementation emerges as a promising approach for addressing sarcopenia and fostering healthy aging among older adults. Furthermore, these results offer a foundation for the development of clinical trials designed to broaden treatment options for individuals at risk of or already experiencing sarcopenia. Such initiatives promise to improve health outcomes and enhance the quality of life for the growing aging population.

## Figures and Tables

**Table 1:** Population’s characteristics.

Characteristic *	Males	Females	Overall	*p*-Value **
	(n = 311)	(n = 408)	(n = 719)	
Age	70.54 (0.26)	70.54 (0.25)	70.54 (0.26)	0.812
BMI (kg/m^2^)	26.3 (3.9)	25.9 (4.7)	26.1 (4.4)	0.257
BMI class				
Less than 18.5	1 (0.3%)	9 (2.2%)	10 (1.4%)	0.001
18.5–25	121 (39%)	195 (48%)	316 (44%)	
25–30	144 (46%)	136 (33%)	280 (39%)	
30 and above	45 (14%)	68 (17%)	113 (16%)	
Handgrip strength (kPa)	87 (15)	74 (14)	80 (16)	<0.001
Handgrip strength (kPa) class				
Low	281 (90%)	366 (90%)	647 (90%)	0.774
Normal	30 (9.6%)	42 (10%)	72 (10%)	
Gait speed 30 m (m/s)	1.32 (0.17)	1.30 (0.18)	1.31 (0.17)	0.101
Gait speed 30 m (m/s) class				
Low	5 (1.6%)	4 (1.0%)	9 (1.3%)	0.511
Normal	306 (98%)	404 (99%)	710 (99%)	
Appendicular Lean Soft Tissue (kg/m^2^)	7.83 (0.77)	6.21 (0.64)	6.91 (1.07)	<0.001
Muscle mass by Appendicular Lean Soft Tissue (kg/m^2^)				
Low	269 (86%)	362 (89%)	631 (88%)	0.366
Normal	42 (14%)	46 (11%)	88 (12%)	
Sarcopenia probable				
No	281 (90%)	366 (90%)	647 (90%)	0.774
Yes	30 (9.6%)	42 (10%)	72 (10%)	
Sarcopenia confirmed				
No	300 (96%)	403 (99%)	703 (98%)	0.037
Yes	11 (3.5%)	5 (1.2%)	16 (2.2%)	
Sarcopenia severe				
No	300 (96%)	403 (99%)	703 (98%)	0.037
Yes	11 (3.5%)	5 (1.2%)	16 (2.2%)	

* Mean (SD); n (%). ** Welch Two Sample *t*-test; Fisher’s exact test; Pearson’s Chi-squared test.

**Table 2:** Population’s nutrient intake.

Characteristic *	Males	Females	Overall	*p*-Value **
	(n = 311)	(n = 408)	(n = 719)	
Energy kcal	2367 (510)	1986 (433)	2151 (504)	<0.001
Protein (g)	95 (22)	82 (20)	88 (21)	<0.001
Fat (g)	96 (27)	83 (26)	88 (27)	<0.001
Carbohydrates (g)	239 (66)	199 (55)	216 (64)	<0.001
Fibers (g)	27 (9)	25 (8)	26 (8)	<0.001
Vitamin C (mg)	149 (79)	151 (73)	150 (76)	0.759
Iron (mg)	13.1 (3.3)	11.5 (3.4)	12.2 (3.4)	<0.001
Calcium (mg)	1069 (417)	1016 (370)	1039 (392)	0.078
Retinol equivalent (µg)	1263 (787)	1048 (543)	1141 (668)	<0.001
Vitamin D (µg)	9.7 (4.6)	8.3 (3.6)	8.9 (4.1)	<0.001
Vitamin E (mg)	16 (12)	16 (15)	16 (14)	0.782
Thiamine (mg)	1.49 (0.38)	1.29 (0.35)	1.38 (0.38)	<0.001
Riboflavin (mg)	1.90 (0.57)	1.67 (0.50)	1.77 (0.54)	<0.001
Niacin equivalent (mg)	41 (9)	35 (8)	38 (9)	<0.001
Vitamin B6 (mg)	2.45 (0.82)	2.26 (0.90)	2.34 (0.87)	0.003
Vitamin B12 (µg)	7.84 (3.92)	6.45 (2.92)	7.05 (3.45)	<0.001
Phosphorus (mg)	1680 (413)	1482 (387)	1568 (410)	<0.001
Magnesium (mg)	419 (103)	374 (101)	394 (104)	<0.001
Potassium (mg)	3880 (962)	3513 (809)	3672 (897)	<0.001
Zinc (mg)	12.41 (2.98)	10.70 (2.76)	11.44 (2.98)	<0.001
Alcohol (g)	16 (16)	9 (10)	12 (14)	<0.001
Saturated fatty acids (g)	38 (13)	33 (13)	35 (13)	<0.001
Monounsaturated fatty acids (g)	35 (10)	30 (9)	32 (10)	<0.001
Polyunsaturated fatty acids (g)	14.9 (5.9)	13.2 (5.6)	13.9 (5.8)	<0.001
EPA (Fatty acid 20:5) (g)	0.26 (0.20)	0.22 (0.16)	0.24 (0.18)	0.014
DHA (Fatty acid 22:6) (g)	0.61 (0.45)	0.54 (0.37)	0.57 (0.41)	0.028
Folate (µg)	359 (108)	345 (107)	351 (107)	0.083
Selenium (µg)	57 (18)	52 (16)	54 (17)	<0.001

* Mean (SD); n (%). ** Welch Two Sample *t*-test; Fisher’s exact test; Pearson’s Chi-squared test.

## Data Availability

The data presented in this study are available on request from the principal investigator.

## Supplementary Materials

The following supporting information can be downloaded at: https://www.mdpi.com/article/10.3390/nu16040568/s1, Figure S1: Population selection; Table S1: Linear association models.
