# Supplementary material for: Nutrient Intake and Its Association with Appendicular Total Lean Mass and Muscle Function and Strength in Older Adults: A Population-Based Study
Source: Nutrients. 2024 Feb 19;16(4):568. doi: 10.3390/nu16040568 (PMC10892025; doi:10.3390/nu16040568)
Supplement: Supplementary file 1 [file nutrients-16-00568-s001.zip › nutrients-2832569-supplementary.pdf]

Figure S1: Sample selection

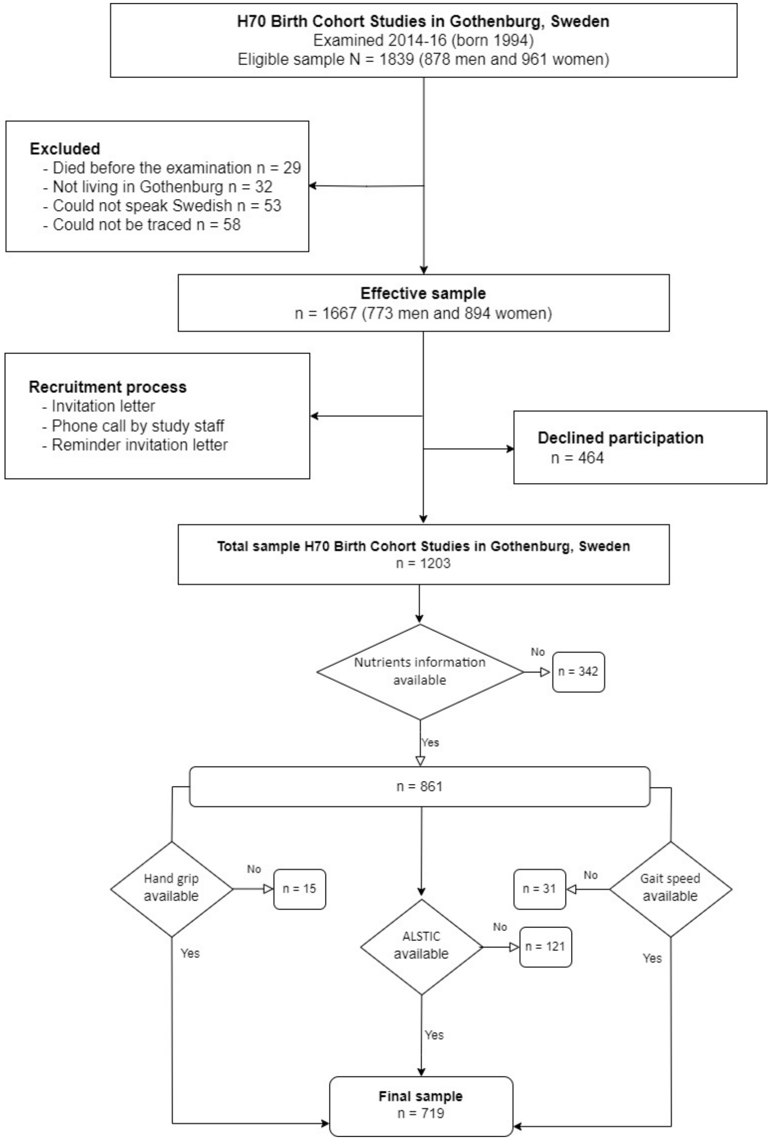

Table S1: Linear association models.

Variable	Appendicular Lean Soft Tissue (kg/m2)					Handgrip strength (kPa)					Gaitspeed 30 m. (m/s)				
	Estimate	Std. Error	P value	P.adjust	Spearman's rho	Estimate	Std. Error	P value	P.adjust	Spearman's rho	Estimate	Std. Error	P value	P.adjust	Spearman's rho
Protein (g)	0,0107	0,0023	0,000	0,000	0.330	0,0721	0,0486	0,139	0,416	0.198	-0,0004	0,0006	0.506	0,416	0.062
Fat (g)	0,0021	0,0022	0,336	0,382	0.248	-0,0213	0,0443	0,630	0,797	0.107	-0,0005	0,0005	0.350	0,797	0.056
Carbohydrates(g)	-0,0015	0,0008	0,067	0,107	0.240	-0,0006	0,0170	0,972	0,981	0.150	-0,0002	0,0002	0.288	0,981	0.047
Fibers(g)	0,0063	0,0048	0,186	0,251	0.113	0,2197	0,0977	0,025	0,223	0.138	0,0021	0,0012	0.076	0,223	0.145
Vitamin C(mg)	0,0008	0,0005	0,122	0,174 †	-0.043	0,0016	0,0105	0,881	0,981	-0.009	0,0001	0,0001	0.482	0,981	0.129
Iron (mg)	0,0439	0,0137	0,001	0,005 †	0.240	0,5741	0,2820	0,042	0,269	0.191	0,0031	0,0034	0.364	0,269	0.114
Calcium(mg)	0,0000	0,0001	0,862	0,862	0.077	0,0013	0,0023	0,586	0,797	0.047	-0,0001	0,0000	0.016	0,797	-0.003
Retinol equivalent (µg)	0,0001	0,0001	0,008	0,017	0.167	0,0005	0,0011	0,675	0,797	0.074	0,0000	0,0000	0.663	0,797	0.003
Vitamin D(µg)	0,0292	0,0089	0,001	0,004	0.219	-0,0060	0,1838	0,974	0,981	0.095	0,0007	0,0022	0.742	0,981	0.075
Vitamin E(mg)	0,0032	0,0034	0,340	0,382	0.111	0,2116	0,0687	0,002	0,058 †	0.039	-0,0002	0,0008	0.810	0,058	0.079
Thiamine(mg)	0,2178	0,1240	0,079	0,119	0.267	2,4390	2,5400	0,337	0,759	0.178	-0,0197	0,0308	0.524	0,759	0.060
Riboflavin(mg)	0,2068	0,0838	0,014	0,029	0.229	1,1740	1,7240	0,496	0,797	0.125	-0,0538	0,0208	0.010	0,797 †	0.045
Niacin equivalent (mg)	0,0176	0,0049	0,000	0,003	0.367	0,0523	0,1023	0,609	0,797	0.220	0,0008	0,0012	0.510	0,797	0.094
Vitamin B6 (mg)	0,0957	0,0515	0,064	0,107	0.175	2,6402	1,0537	0,012	0,167	0.127	0,0120	0,0128	0.347	0,167	0.148
Vitamin B12 (µg)	0,0397	0,0104	0,000	0,001	0.231	0,1225	0,2165	0,571	0,797	0.125	-0,0025	0,0026	0.343	0,797	0.023
Phosphorus (mg)	0,0004	0,0001	0,007	0,016	0.263	0,0053	0,0027	0,050	0,269	0.175	0,0000	0,0000	0.423	0,269	0.062
Magnesium(mg)	0,0005	0,0005	0,280	0,360	0.220	0,0098	0,0095	0,302	0,741	0.163	0,0002	0,0001	0.057	0,741	0.128
Potassium(mg)	0,0001	0,0000	0,027	0,048	0.212	0,0004	0,0010	0,665	0,797	0.154	0,0000	0,0000	0.223	0,797	0.124
Zinc (mg)	0,0604	0,0172	0,000	0,003	0.302	0,6027	0,3556	0,091	0,349	0.189	-0,0079	0,0043	0.066	0,349	0.034
Alcohol(g)	-0,0017	0,0024	0,489	0,528	0.165	0,0207	0,0499	0,679	0,797	0.108	0,0005	0,0006	0.370	0,797	0.099
Saturated fatty acids(g)	-0,0019	0,0038	0,626	0,650	0.189	-0,0019	0,0780	0,981	0,981	0.088	-0,0016	0,0009	0.100	0,981	0.049
Monounsaturated fatty acids (g)	0,0053	0,0052	0,308	0,378	0.264	-0,0736	0,1066	0,490	0,797	0.109	-0,0012	0,0013	0.369	0,797	0.042

Polyunsaturated fatty acids (g)	0,0171	0,0072	0,017	0,034	0.190	-0,0775	0,1477	0,600	0,737	0.121	0,0017	0,0018	0.343	0,737	0.090
EPA (Fatty acid 20:5) (g)	0,5892	0,1968	0,003	0,008	0.117	4,4431	4,0505	0,273	0,797	0.080	0,0289	0,0491	0.557	0,797	0.105
DHA (Fatty acid 22:6) (g)	0,2978	0,0875	0,001	0,003	0.125	0,8009	1,8062	0,658	0,797	0.068	0,0195	0,0219	0.372	0,797	0.087
Folate (µg)	0,0012	0,0004	0,002	0,006 †	0.087	0,0048	0,0080	0,548	0,269	0.089	0,0001	0,0001	0.175	0,269	0.157
Selenium (µg)	0,0109	0,0024	0,000	0,000	0.227	0,0925	0,0491	0,060	0,416	0.124	0,0002	0,0006	0.712	0,416	0.092

† Significant interaction with sex
